# Association between serum 25-hydroxy vitamin D level and menstrual cycle length and regularity: A cross-sectional observational study

**DOI:** 10.18502/ijrm.v19i11.9913

**Published:** 2021-12-13

**Authors:** Vinita Singh, Neelam Tamar, Zamir Lone, Esha Das, Rajshree Sahu, Sagarika Majumdar

**Affiliations:** Obstetrics and Gynecology Department, Aiims, Raipur, India.

**Keywords:** Menstrual disturbances, Ovulation, Reproductive period, Vitamin D deficiency, Dietary supplements, Polycystic ovarian syndrome.

## Abstract

**Background:**

Research examining the role of vitamin D deficiency and the development of menstrual disorders in women is of widespread interest. Studies have been published showing that supplementation with high-dose vitamin D can lead to the restoration of the menstrual cycle. We lack adequate information regarding the effect of vitamin D levels on the physiology of menstruation and further on fertility in women of reproductive age due to the contradictory results reported by studies.

**Objective:**

To study the association of 25-hydroxy vitamin D with menstrual cycle characteristics including long and short cycle length and cycle irregularity.

**Materials and Methods:**

In this cross-sectional study, serum vitamin D levels of 166 women attending an outpatient department with menstrual irregularities after excluding all obvious causes of menstrual disorders (n = 83) between April-June 2019 were measured and were compared with women of similar profiles with complaints other than menstrual irregularities (n = 83).

**Results:**

A decreased level of vitamin D was associated with a 13.3 times odds of an irregular cycle (OR (95% CI): 13.30 (5.79-30.60), p < 0.001). 25-hydroxy vitamin D was not associated with age or body mass index. We found a significant difference (p < 0.001) in mean vitamin D levels among the females with irregular cycles vs. regular cycles.

**Conclusion:**

Vitamin D plays a role in the physiology of reproduction specific to the menstrual cycle and ovulation. Long-term prospective studies assessing the exact cutoff value and the exact dose of supplementation required are needed.

## 1. Introduction 

It is already well known that vitamin D plays an important role in maintaining bone strength by controlling the mineralization of bones. There is growing evidence of vitamin D also being involved in cell proliferation and immune regulation (1, 2). Vitamin D tends to exert its diversified role in influencing reproductive physiology through vitamin D receptors, which are found in the female reproductive tract, such as in the ovaries (particularly in the granulosa cells), uterus, and placenta (3). Vitamin D has been documented in patients with polycystic ovarian syndrome (PCOS), hyperandrogenism and infertility (4, 5). Vitamin D likely executes its control on ovarian function and hence the menstrual cycle through anti-mullerian hormone (AMH) receptors, which also share a domain for the vitamin D response element (3, 6). Vitamin D supplementation in a high dose can correct metabolic disorders associated with PCOS (7, 8). Studies have shown that vitamin D can play a role in ovarian reserve and dysmenorrhoea (9, 10). Vitamin D and calcium supplementation in PCOS patients has also been shown to influence follicle maturation and menstruation (11, 12). Some researchers question the role of vitamin D (in combination with calcium) in any kind of menstrual problems in females (13). Altered calcium homeostasis and parathormone PTH levels secondary to vitamin D deﬁciency could also be the cause of the dysregulated follicular development causing menstrual dysfunction in women with PCOS (4, 14).

This study was done to find whether any association exists between deficient vitamin D levels and menstrual irregularities. Vitamin D is inexpensive and easily available with minimal side effects, so it can be considered for supplementation in women with menstrual irregularities if any positive association is found.

## 2. Materials and Methods 

We conducted this cross-sectional observational study in a tertiary care teaching hospital located in central India over three months from April 2019 to June 2019. Female patients of reproductive age attending the gynecology outpatient department at Aiims Raipur with complaints of menstrual irregularities were included. The sample size was calculated based on standard deviation and minimum difference detected for the mean vitamin D levels between irregular and regular cycles taken from a previous study (7), with 80% power and a 5% level of significance. The necessary sample size was calculated as 166 (83 in each group).

The inclusion criteria were: female patients of reproductive age attending the gynecology outpatient department at Aiims Raipur with complaints of menstrual irregularity (defined as a shortest to longest cycle variation of ≥ 8-10 days). Participants were divided into two groups: (i) frequent cycle ( < 24 days interval); (ii) infrequent cycle ( > 38 days). The exclusion criteria were: 1) known etiology for irregular menstruation like thyroid disorder, hyper prolactinoma, uterine pathology, or ovarian tumor; 2) patients on hormonal therapy, insulin sensitizers, vitamin D, calcium, or glucocorticoids. Women with similar profiles attending the gynecology outpatient department with complaints other than menstrual irregularities were recruited as controls.

The case records form was filled for all the participants, which included demographic details of the recruited patients. A detailed history was taken regarding their menstrual cycle length, amount of flow, pain associated with menses or any other associated pathology. A detailed medical, surgical, family, and personal history was taken, along with a dietary history (with special reference to the intake of calcium-rich food such as milk, cheese, paneer and dark green leafy vegetables), and drug history (e.g., hormonal supplementation, vitamin supplementation, metformin, glucocorticoids, beta-blockers).

A detailed general examination including body mass index (BMI) was calculated for each participant. About 5 ml of venous blood was withdrawn from the antecubital vein of participants in a plain red vacutainer and serum 25-hydroxy vitamin D (25(OH)D) levels were measured. Vitamin D in the serum was measured using the ADVIA centaur vitamin D assay on the ADVIA Centaur XP system by competitive immunoassay, which used an anti-fluorescein monoclonal mouse antibody of 25(OH)D.

### Ethical considerations

The study was started only after obtaining ethical approval from the Aiims Ethical Committee (Code: AIIMSRPR/IEC/2018/155) and obtaining informed consent from all the participants.

### Statistical analysis

Statistical analysis was carried out using the Statistical Package for the Social Sciences (SPSS, version 16.0 for Windows, SPSS Inc., Chicago, IL, USA). Continuous and categorical variables were expressed as mean (standard deviation) and frequency (%), respectively. 25(OH)D was structured both as a continuous linear variable and as a dichotomous variable of “insufficient” vs. “sufficient”, based on the World Health Organization's cutoff of 20 ng/ml (15). Chi-square test was done to determine the difference in proportions of demographic data such as age, BMI, marital history, and parity. Student's *t* test was applied to compare the mean vitamin D levels between women with irregular vs. regular cycles and long vs. short cycles. Associations between 25(OH)D and regular and irregular cycles, age and BMI were estimated through logistic regression. Two-sided p-values were considered significant at p < 0.05.

## 3. Results

**Figure 1:**
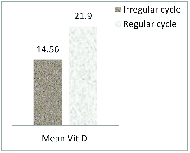
Comparison of mean vitamin D levels in women with irregular vs. regular cycles.

Table I reflects the sociodemographic characteristics of the females in the two study groups and it was observed that there was no significant difference between the two groups in terms of age, BMI or marital status but there was a significant difference in terms of parity in the two groups (p = 0.02). Table II and figure 1 show that there was a significant difference (p < 0.001) in mean vitamin D levels between those that had irregular cycles vs. regular cycles. The women who had regular cycles had significantly higher vitamin D levels than those with irregular cycles. There was no significant difference (p > 0.05) in the mean vitamin D levels between the females who had infrequent cycles vs. those with frequent cycles.

Table III shows the associations between vitamin D levels and cycle regularity, age, and BMI. 86.7% of patients with menstrual irregularities were vitamin D deficient, i.e. they had a vitamin D level < 20 ng/ml, whereas only 31.0% of patients with normal menstruation were found to have vitamin D deficiency. Among patients with menstrual disorders who were vitamin D deficient, 83.1% of them presented with infrequent cycles, 16.9% had frequent cycles and 25.0% suffered from dysmenorrhoea. Binary logistic regression was applied. Decreased levels of vitamin D were associated with a 13.3 times higher odds of irregular cycles (odds ratio: 13.3 (confidence interval: 5.79, 30.6), p < 0.001). 25(OH)D levels were not associated with age or BMI.

**Table 1:** Sociodemographic characteristics of the females


**Variables**	**Cases (n = 83)**	**Controls (n = 83)**	**p-value**
** Age (yr)**			
**18-21 **	10 (12.05)	16 (19.30)	
**22-29 **	47 (56.63)	50 (60.23)	
** > 30 **	26 (31.32)	17 (20.47)	Chi-square = 3.36 df = 2 p = 0.18
** BMI (kg/m ${}^{2}$ )**			
** < 18.5 (underweight)**	4 (4.81)	7 (8.43)		
**18.5-24.9 (normal)**	36 (43.38)	32 (38.55)	
**25-29.9 (overweight)**	35 (42.17)	15 (18.07)	
** ≥ 30 (obese)**	8 (9.64)	4 (4.81)	Chi-square = 6.15 df = 3 p = 0.10
** Marital history**			
**Married**	53 (17.66)	54 (65.06)		
**Unmarried**	30 (36.14)	29 (34.94)		Chi-square = 0.26 df = 1 p = 0.87
** Parity**				
**Nullipara**	38 (45.79)	49 (59.04)	
**Primipara**	19 (22.89)	23 (27.71)	
**Multipara**	26 (31.32)	11 (13.25)	Chi-square = 7.85 df = 2 p = 0.02*
Data presented as n (%). BMI: Body mass index, Df: Degrees of freedom, *P < 0.05 so is significant			

**Table 2:** Comparison of vitamin D levels based on regularity and duration of the cycle


	**N**	**Mean ± SD**	*t* **value**	**p-value**
**Irregular cycle**	83	14.56 ± 8.76	
**Regular cycle**	83	21.90 ± 6.15	-6.27	≤ 0.001**
**Infrequent cycle **	69	15.00 ± 9.17			
**Frequent cycle**	14	12.96 ± 6.13	1.10	0.27***
Student's *t* test, **P < 0.001 so is significant, *** P > 0.05 so is not significant				

**Table 3:** Association of vitamin D levels with regularity of cycle, age, and BMI


		**N (%)**	25-hydroxy vitamin D*	**OR (95% CI)**	**p-value**
** < 20 ng/ml**		96 (57.1)	-
** ≥ 20 ng/ml**		72 (42.9)	-
** Irregular cycle**					
	**No**	83 (50.0)	21.90 ± 6.15	13.32 (5.79-30.61)	≤ 0.001**
	**Yes**	83 (50.0)	14.56 ± 8.76	
** Age group (yr)**					
	**18-21**	16 (9.5)	16.57 ± 8.63	0.23
	**22-29**	102 (60.7)	18.77 ± 7.71	0.85 (0.13-5.34)	0.86
	**30-39**	34 (20.2)	18.08 ± 11.22	2.28 (0.54-9.50)	0.26
	** ≥ 40**	16 (9.5)	16.80 ± 5.06	3.53 (0.69-18.09)	0.13
** BMI (kg/m ${}^{2}$ ) group**					
	** < 18.5 (underweight)**	13 (7.7)	19.61 ± 5.54	0.16
	**18.5-24.9 (normal)**	79 (47.0)	19.55 ± 8.46	3.39 (0.54-21.32)	0.19
	**25-29.9 (overweight)**	61 (36.3)	16.40 ± 8.34	4.55 (1.04-19.75)	0.04
	** ≥ 30 (obese)**	15 (8.9)	17.58 ± 9.35	2.42 (0.52-11.19)	0.26
*Data presented as Mean ± SD. BMI: Body mass index, **P < 0.001 so is highly significant. Association estimated through binary logistic regression and results expressed in p-values					

## 4. Discussions

Our study's results demonstrated a significant difference (p < 0.001) in mean vitamin D levels in females with irregular cycles vs. in those with regular cycles. The women with regular cycles had significantly higher vitamin D levels. A lower level of vitamin D was associated with 13.3 times odds of having an irregular cycle (OR (95% CI): 13.3 (5.79-30.6), p < 0.001).

Several other studies have similarly found that patients with PCOS were vitamin D deficient (4, 5, 16). In our study, there was no significant difference (p > 0.05) in mean vitamin D levels among the females with long cycles vs. those with short cycles. Previous similar studies have reported associations between lower levels of 25(OH)D and an increased odds of either irregular or long cycles (9).

Lower levels of 25(OH)D were associated with both long menstrual cycles and long follicular phases, and with a tendency towards short luteal phases. The strongest associations were for vitamin D deficiency ( < 20 ng/ml). The probability of a long follicular phase and the probability of a short luteal phase both increased with decreasing 25(OH)D (17).

No difference was found between the PCOS group and control group, i.e. 80% vs. 70%, respectively, had vitamin D deficiency (p = 0.14) (18). In a cross-sectional study on reproductive age African American women it was concluded that insufficient 25(OH)D levels were associated with a prolonged follicular phase leading to delayed ovulation and hence long menstrual cycles (but not short or irregular cycles) (9). Other studies have focused on supplementing vitamin D and calcium along with other drugs like metformin to improve ovulation and menstrual disorders in PCOS patients (19, 20). In our study, no association was seen between age or BMI and vitamin D levels, but there are studies in which subjects diagnosed with PCOS showed improvement in their complaints of infertility, BMI, and biochemical and metabolic disorders after vitamin D and calcium supplementation (13, 21). Several other studies along with one randomized, single-blind, placebo-controlled intervention study have found evidence of improvement in menstrual regularity after calcium and vitamin D supplementation along with metformin (p = 0.002) (4, 22). Dietary supplementation with vitamin D through fortified foods or intake of vitamin D sachets containing 60,000 units along with calcium in females with vitamin D deficiency could be an important step in improving their reproductive health (23, 24). Vitamin D deficiency is very common in women of reproductive age which may affect menstrual cycle length due to a pronged follicular phase causing delayed ovulation. This is an emerging field of research and hence further prospective studies with larger sample sizes and longer duration are needed to examine the role of vitamin D in reproduction specific to the menstrual cycle and fertility of reproductive age women, the underlying mechanisms, effective cutoff values, and exact dose of supplementation required (25). The current study had the limitation of a small sample size; low levels of vitamin D are generally found in the local population so to reach any conclusive evidence and provide recommendations we would require a large sample size with properly matched cases and controls from the population.

## 5. Conclusion

Menstrual irregularities in reproductive age women is a very common problem in our society and vitamin D deficiency is also highly prevalent. Other studies have concluded that the optimum blood level of vitamin D has a role in folliculogenesis, normal ovulation function and regular menstruation. This study was conducted to examine whether any association exists between deficient vitamin D levels and menstrual irregularities. Vitamin D is inexpensive and easily available with minimal side effects, so it can be considered for supplementation in women with menstrual irregularities. Further studies are recommended for reaching more conclusive results regarding the effectiveness of vitamin D supplementation for the correction of menstrual irregularities and in treatment of infertility, and its other benefits.

## Conflict of Interest

There is no conflict of interest in the present study.
